# Regulation of ClC-2 Chloride Channel Proteostasis by Molecular Chaperones: Correction of Leukodystrophy-Associated Defect

**DOI:** 10.3390/ijms22115859

**Published:** 2021-05-30

**Authors:** Ssu-Ju Fu, Meng-Chun Hu, Cheng-Tsung Hsiao, An-Ting Cheng, Tsung-Yu Chen, Chung-Jiuan Jeng, Chih-Yung Tang

**Affiliations:** 1 Department of Physiology, College of Medicine, National Taiwan University, Taipei 10051, Taiwan; d01441001@ntu.edu.tw (S.-J.F.); mengchun@ntu.edu.tw (M.-C.H.); hsiaoct26@gmail.com (C.-T.H.); r08441010@ntu.edu.tw (A.-T.C.); 2 Institute of Anatomy and Cell Biology, College of Medicine, National Yang Ming Chiao Tung University, Taipei 12212, Taiwan; 3 Department of Neurology, Taipei Veterans General Hospital, Taipei 12217, Taiwan; 4 Center for Neuroscience and Department of Neurology, University of California, Davis, CA 95616, USA; tycchen@ucdavis.edu; 5 Brain Research Center, National Yang Ming Chiao Tung University, Taipei 12212, Taiwan; 6 Graduate Institute of Brain and Mind Sciences, College of Medicine, National Taiwan University, Taipei 10051, Taiwan

**Keywords:** proteostasis, channelopathy, protein quality control, chaperone, co-chaperone, 17-AAG

## Abstract

The ClC-2 channel plays a critical role in maintaining ion homeostasis in the brain and the testis. Loss-of-function mutations in the ClC-2-encoding human *CLCN2* gene are linked to the white matter disease leukodystrophy. *Clcn2*-deficient mice display neuronal myelin vacuolation and testicular degeneration. Leukodystrophy-causing ClC-2 mutant channels are associated with anomalous proteostasis manifesting enhanced endoplasmic reticulum (ER)-associated degradation. The molecular nature of the ER quality control system for ClC-2 protein remains elusive. In mouse testicular tissues and Leydig cells, we demonstrated that endogenous ClC-2 co-existed in the same protein complex with the molecular chaperones heat shock protein 90β (Hsp90β) and heat shock cognate protein (Hsc70), as well as the associated co-chaperones Hsp70/Hsp90 organizing protein (HOP), activator of Hsp90 ATPase homolog 1 (Aha1), and FK506-binding protein 8 (FKBP8). Further biochemical analyses revealed that the Hsp90β-Hsc70 chaperone/co-chaperone system promoted mouse and human ClC-2 protein biogenesis. FKBP8 additionally facilitated membrane trafficking of ClC-2 channels. Interestingly, treatment with the Hsp90-targeting small molecule 17-allylamino-17-demethoxygeldanamycin (17-AAG) substantially boosted ClC-2 protein expression. Also, 17-AAG effectively increased both total and cell surface protein levels of leukodystrophy-causing loss-of-function ClC-2 mutant channels. Our findings highlight the therapeutic potential of 17-AAG in correcting anomalous ClC-2 proteostasis associated with leukodystrophy.

## 1. Introduction

The ubiquitously expressed ClC-2 chloride (Cl^−^) channel is activated by membrane hyperpolarization and osmotic cell swelling, and plays an essential role in the regulation of Cl^−^ homeostasis in a wide variety of different tissues [1,2,3,4,5,6,7]. In mice, knockout of the *Clcn2* gene, which encodes the voltage-gated ClC-2 channel, results in neuronal fluid accumulation and myelin vacuolation in the brain, as well as substantial degeneration of the retina and the testis [8,9,10]. In humans, loss-of-function mutations in the *CLCN2* gene are associated with a subtype of the white matter disorder leukodystrophy, also known as *CLCN2*-related leukoencephalopathy, manifesting as intramyelinic edema in the brain and perhaps infertility [11,12,13,14]. Along with other studies in different types of *Clcn2*-deficient mice [15,16], these findings support the notion that the ClC-2 channel is important for extracellular ion homeostasis in the brain and the testis.

Patients with *CLCN2*-related leukoencephalopathy present with homozygous or compound-heterozygous mutations, indicating that this rare disorder is primarily caused by autosomal recessive mutations in the *CLCN2* gene [11,12,14,17,18,19]. To date, more than 10 disease-related *CLCN2* mutations have been identified, including missense, insertion, and deletion mutations. Functional and biochemical analyses show that, in addition to altered voltage-dependent gating property and reduced Cl^−^ current amplitude, leukodystrophy-causing ClC-2 mutant channels are associated with defective protein stability and impaired membrane trafficking [11,13,20], consistent with the presence of disease-related anomalous ClC-2 protein homeostasis (proteostasis). Proteostasis is largely maintained by multiple translational and post-translational mechanisms, thereby ensuring appropriate conformation, stability, and subcellular localization of proteins [21,22]. One of the crucial proteostasis mechanisms for membrane proteins entails selective removal of misfolded proteins from the endoplasmic reticulum (ER) and subsequent degradation by the proteasome, commonly referred to as ER-associated degradation [23,24].

ER-associated degradation comprises a series of stringent protein quality control systems, as well as concerted activity of ubiquitination machinery [24,25,26]. Elucidation of the interplay between ClC-2-related ER protein quality control systems and proteasomal degradation pathways is therefore essential for addressing the molecular pathophysiology of leukodystrophy. We demonstrated recently that the cullin 4 (CUL4)-damage-specific DNA binding protein 1 (DDB1)-cereblon (CRBN) E3 ubiquitin ligase complex promotes ubiquitination and ER-associated degradation of wild-type (WT) and disease-related mutant ClC-2 channels [20]. The molecular nature of the ER quality control system for ClC-2 protein, however, is still unclear.

A cardinal process during ER protein quality control involves conformation surveillance of nascent polypeptides by a network of molecular chaperones and cofactors (co-chaperones) that proficiently facilitate protein folding, thereby minimizing degradation of misfolded proteins [27,28,29]. Interestingly, the interconnected heat shock protein 70 (Hsp70) and heat shock protein 90 (Hsp90) molecular chaperone systems, along with their associated co-chaperones such as Hsp70/Hsp90 organizing protein (HOP), activator of Hsp90 ATPase homolog 1 (Aha1), and FK506-binding protein 8 (FKBP8), have been demonstrated to play essential roles in ER quality control of several types of Cl^−^ channels [30,31,32,33,34]. In the current study, we set out to identify the chaperone/co-chaperone network monitoring endogenous ClC-2 proteostasis in mouse testicular tissues and Leydig cells. By employing heterologous expression in the human embryonic kidney (HEK) 293T cells, we also explored the therapeutic potential of correcting leukodystrophy-associated anomalous human ClC-2 proteostasis by modulating chaperone/co-chaperone activity.

## 2. Results

### 2.1. Association of Endogenous ClC-2 with Chaperones and Co-Chaperones in Native Tissue

**Figure 1:**
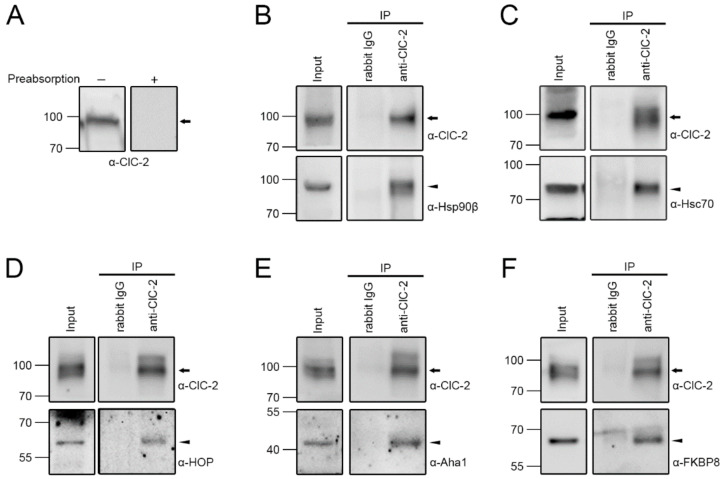
Interaction of molecular chaperones and co-chaperones with endogenous ClC-2 in mouse testes. (**A**) Endogenous ClC-2 protein signal in mouse testes. The specificity of the rabbit-derived anti-ClC-2 antibody (α-ClC-2) was verified by preabsorption with a control antigen peptide. (**B**–**F**) Co-immunoprecipitation of endogenous Hsp90β (**B**), Hsc70 (**C**), HOP (**D**), Aha1 (**E**), or FKBP8 (**F**) with ClC-2. Mouse testis lysates were immunoprecipitated (IP) with the rabbit IgG or α-ClC-2. The molecular weight markers (in kilodaltons) and immunoblotting antibodies (α-Hsp90β, α-Hsc70, α-HOP, α-Aha1, and α-FKBP8) are labeled to the left and right, respectively. The protein bands corresponding to endogenous ClC-2 and chaperones/co-chaperones are highlighted with the black arrow and the black arrowhead, respectively. Corresponding expression levels of ClC-2 and chaperones/co-chaperones in the lysates are shown in the Input lane. In all cases hereafter, input represents about 10% of the total protein used for immunoprecipitation.

Previous biochemical evidence supports the interaction between the ClC-2 Cl^−^ channel and the molecular chaperone Hsp90 in the mouse brain [35]. We therefore began by asking whether this well-known molecular chaperone, as well as its associated molecular chaperone and co-chaperones, may interact with endogenous ClC-2 protein profusely expressed in mouse testes (Figure 1A). Consistent with the previous observation in the mouse brain, Figure 1B depicts that endogenous ClC-2 in testes was readily co-immunoprecipitated with Hsp90β, the constitutive Hsp90 isoform. Furthermore, the molecular chaperone heat shock cognate protein 70 (Hsc70), the constitutively expressed Hsp70 isoform, also co-existed in the same protein complex with endogenous ClC-2 (Figure 1C). Our co-immunoprecipitation study further identified HOP, a soluble co-chaperone regulating Hsp90 ATPase activity and mediating the interaction of Hsp70 and Hsp90 [27,36], as a binding partner of endogenous ClC-2 (Figure 1D). Figure 1E illustrates that Aha1, another cytosolic co-chaperone regulating the ATPase activity of Hsp90 [27,36], was also co-immunoprecipitated with endogenous ClC-2. Lastly, the co-immunoprecipitation result in Figure 1F is consistent with the idea that endogenous ClC-2 is associated with the co-chaperone FKBP8 (also known as FKBP38), which is an Hsp90-associated membrane-anchored immunophilin with potential peptidyl-prolyl *cis-trans* isomerase function [37,38].

**Figure 2:**
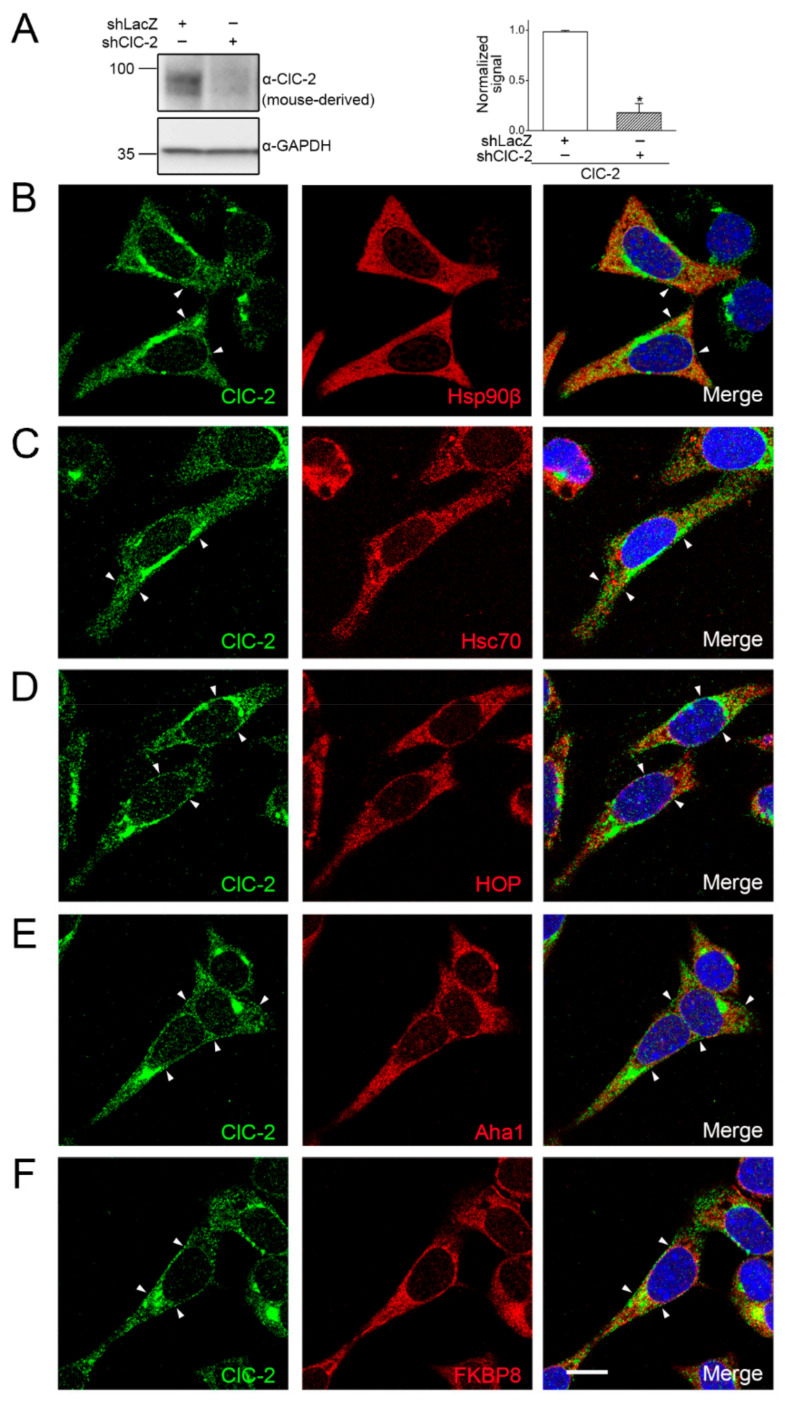
Co-localization of molecular chaperones and co-chaperones with endogenous ClC-2 in mouse MA-10 cells. (**A**) (Left) Endogenous ClC-2 protein signal in mouse MA-10 Leydig cells. The specificity of the mouse-derived anti-ClC-2 antibody was verified by shRNA knock-down of mouse ClC-2 (shClC-2). shRNA knock-down of LacZ (shLacZ) was employed as the control experiment. GAPDH was used as the loading control. (Right) Quantification of relative ClC-2 protein levels. Protein density was standardized as the ratio of the ClC-2 signal to the cognate GAPDH signal. Values from the shClC-2 group (hatched bar) was then normalized to those for the corresponding shLacZ control (clear bar). Asterisk denotes significant difference from the shLacZ control (*, *t* test: *p* < 0.05; *n* = 4). (**B**–**F**) Representative confocal micrographs showing the immunofluorescence staining patterns of endogenous ClC-2 (green) and chaperones/co-chaperones (red). Fixed MA-10 cells were stained with the mouse-derived anti-ClC-2 antibody, as well as rabbit-derived antibodies against the indicated molecular chaperones/co-chaperones, under the permeabilized configuration. Merged images of ClC-2 and chaperone/co-chaperone signals are shown in the rightmost panels, where DAPI (blue) was employed as a nuclear counterstain. Arrowheads denote plasma membrane-localization of ClC-2. Scale bar = 15 μm. Data are representative of at least three independent experiments.

Given that complete knockout of ClC-2 in mice leads to severe testicular degeneration involving abnormal Sertoli cells and hyperplasia of Leydig cells [8,9], we went on to examine the subcellular localization of endogenous ClC-2 and the foregoing chaperones/co-chaperones in a cell line derived from mouse Leydig cells, the MA-10 cell [39,40]. Endogenous ClC-2 protein expression was verified with shRNA knock-down of mouse ClC-2 in MA-10 cells (Figure 2A). As illustrated in Figure 2B, immunofluorescence analyses showed that endogenous ClC-2 channels in MA-10 Leydig cells were abundantly present at the plasma membrane; notably, a prominent portion of endogenous ClC-2 was also present in the perinuclear region and partially co-localized with Hsp90β. Similarly, a notable fraction of cytoplasmic ClC-2 co-localized with endogenous Hsc70, HOP, Aha1, or FKBP8 in MA-10 cells as well (Figure 2C–F). Taken together, both our biochemical and immunofluorescence data support the notion that endogenous ClC-2 channels are associated with the molecular chaperones Hsp90β and Hsc70, as well as their co-chaperones HOP, Aha1, and FKBP8.

### 2.2. Regulation of ClC-2 Protein Expression by Chaperones and Co-Chaperones

**Figure 3:**
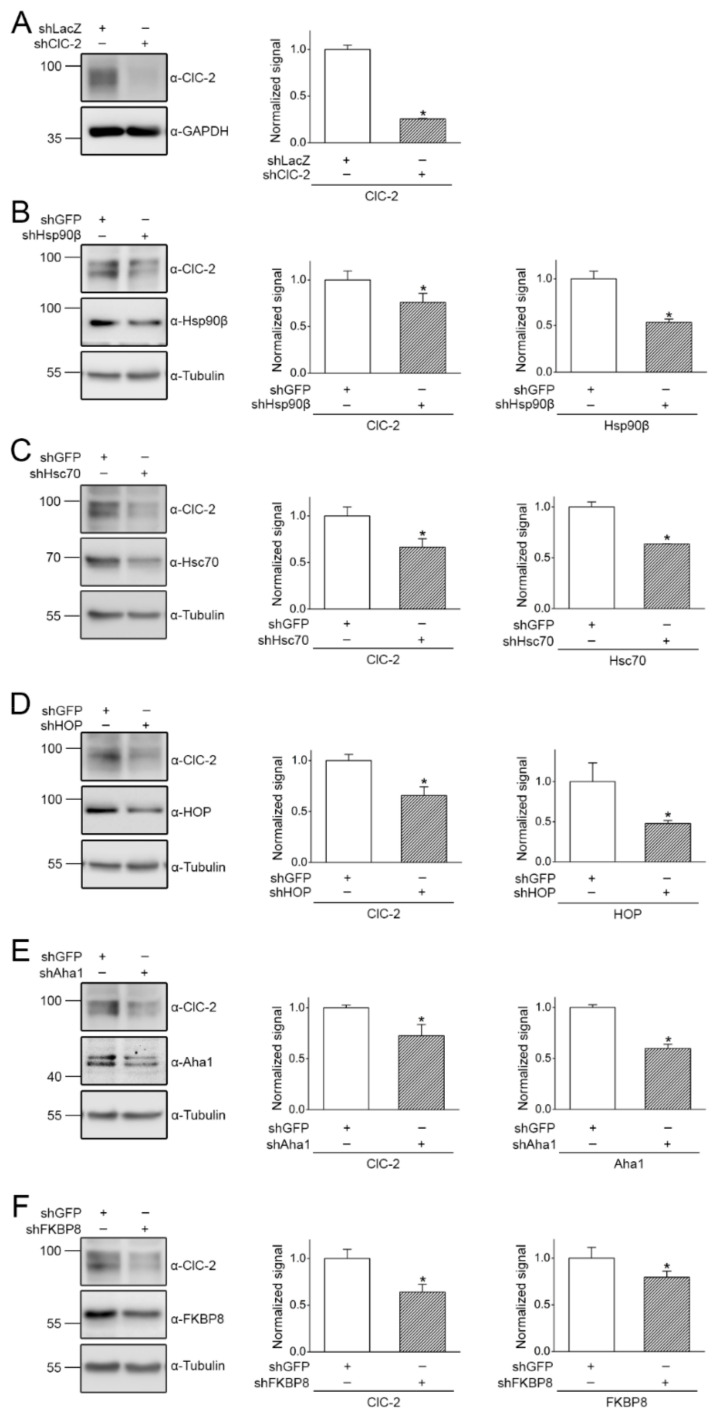
shRNA knock-down of endogenous chaperones or co-chaperones in mouse MA-10 cells. (**A**) Verification of the specificity of the rabbit-derived anti-ClC-2 antibody in mouse MA-10 Leydig cells. (Left) Representative immunoblot showing the effect of shLacZ and shClC-2 knock-down on endogenous ClC-2 protein signal. (Right) Quantification of relative ClC-2 protein levels. Protein density was standardized as the ratio of the ClC-2 signal to the cognate GAPDH signal. Values from the shClC-2 group (hatched bar) was then normalized to those for the corresponding shLacZ control (clear bar). Asterisk denotes significant difference from the shLacZ control (*, *t* test: *p* < 0.05; *n* = 3). (**B**–**F**) (Left panels) Representative immunoblots showing the effect of shRNA knock-down of chaperones/co-chaperones (shHsp90β, shHsc70, shHOP, shAha1, and shFKBP8) on endogenous ClC-2 expression. Infection with shGFP was used as the control experiment. Tubulin was used as the loading control. (Right panels) Quantification of relative ClC-2 and chaperone/co-chaperone protein levels. Protein density was standardized as the ratio of the ClC-2 signal to the cognate tubulin signal. Values from the shRNA knock-down groups (hatched bars) were then normalized to those for the corresponding shGFP control (clear bars). Asterisks denote significant difference from the shGFP control (*, *t* test: *p* < 0.05; *n* = 4–10).

The next question we asked was whether these molecular chaperones and co-chaperones play a role in endogenous ClC-2 protein expression. To this end, we first employed RNA interference with specific shRNA for various chaperones/co-chaperones in mouse MA-10 Leydig cells. The specificity of the rabbit-derived anti-ClC-2 antibody was verified with shRNA knock-down of mouse ClC-2 in MA-10 cells (Figure 3A). Consistent with a regulatory role of Hsp90β, shRNA knock-down of endogenous Hsp90β substantially reduced ClC-2 protein expression in MA-10 cells (Figure 3B). Figure 3C–F further demonstrate that shRNA knock-down of endogenous Hsc70, HOP, Aha1, or FKBP8 decreased ClC-2 protein expression by about 20–36% in MA-10 cells.

**Figure 4:**
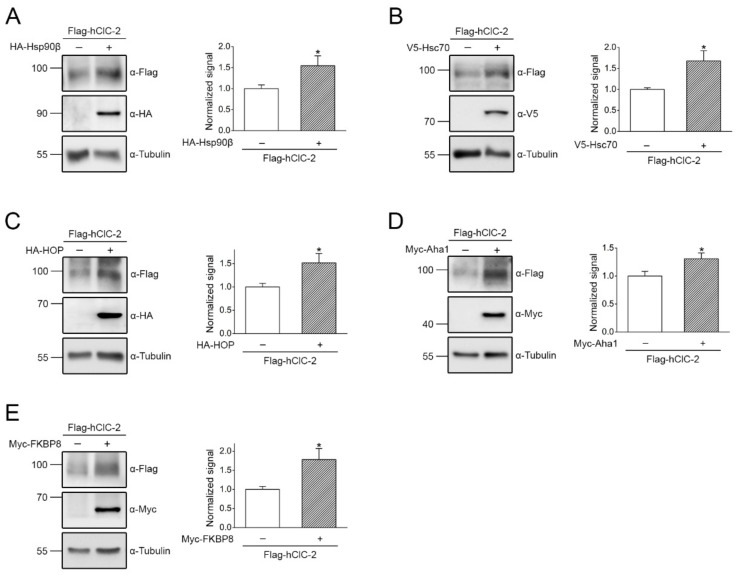
Co-expression with chaperone/co-chaperone increases human ClC-2 protein level in HEK293T cells. Heterologous expression of Flag-tagged human ClC-2 channels (Flag-hClC-2) in HEK293T cells. (Left panels) Representative immunoblots showing the effect of co-expressing HA-tagged Hsp90β (HA-Hsp90β) (**A**), V5-tagged Hsc70 (V5-Hsc70) (**B**), HA-tagged HOP (HA-HOP) (**C**), Myc-tagged Aha1 (Myc-Aha1) (**D**), or Myc-tagged FKBP8 (Myc-FKBP8) (**E**) on Flag-hClC-2 protein expression. cDNA for Flag-hClC-2 was co-transfected with that for the indicated chaperone/co-chaperone in the molar ratio 1:3. Co-expression with the empty vector was employed as the control experiment. Tubulin was used as the loading control. (Right panels) Quantification of relative ClC-2 protein levels in the presence of various chaperones/co-chaperones. Protein density was standardized as the ratio of the ClC-2 signal to the cognate tubulin signal. Values from the chaperone/co-chaperone co-expression groups (hatched bars) were then normalized to those for the corresponding vector control (clear bars). Asterisks denote significant difference from the vector control (*, *t* test: *p* < 0.05; *n* = 5–11).

The results derived from the preceding shRNA knock-down experiments suggest that these molecular chaperones and co-chaperones may promote endogenous ClC-2 protein expression in mouse Leydig cells. To further verify the validity of this inference, we examined the effect of enhancing chaperone/co-chaperone expression on human ClC-2 channels heterologously expressed in HEK293T cells. As estimated from immunofluorescence analyses (data not shown), the cDNA transfection efficiency was about 70–90%. Figure 4A,B show that co-expression with Hsp90β or Hsc70 significantly increased human ClC-2 protein level by more than 50%. Comparable protein enrichment effects were also observed when we co-expressed HOP, Aha1, or FKBP8 with human ClC-2 in HEK293T cells (Figure 4C–E).

**Figure 5:**
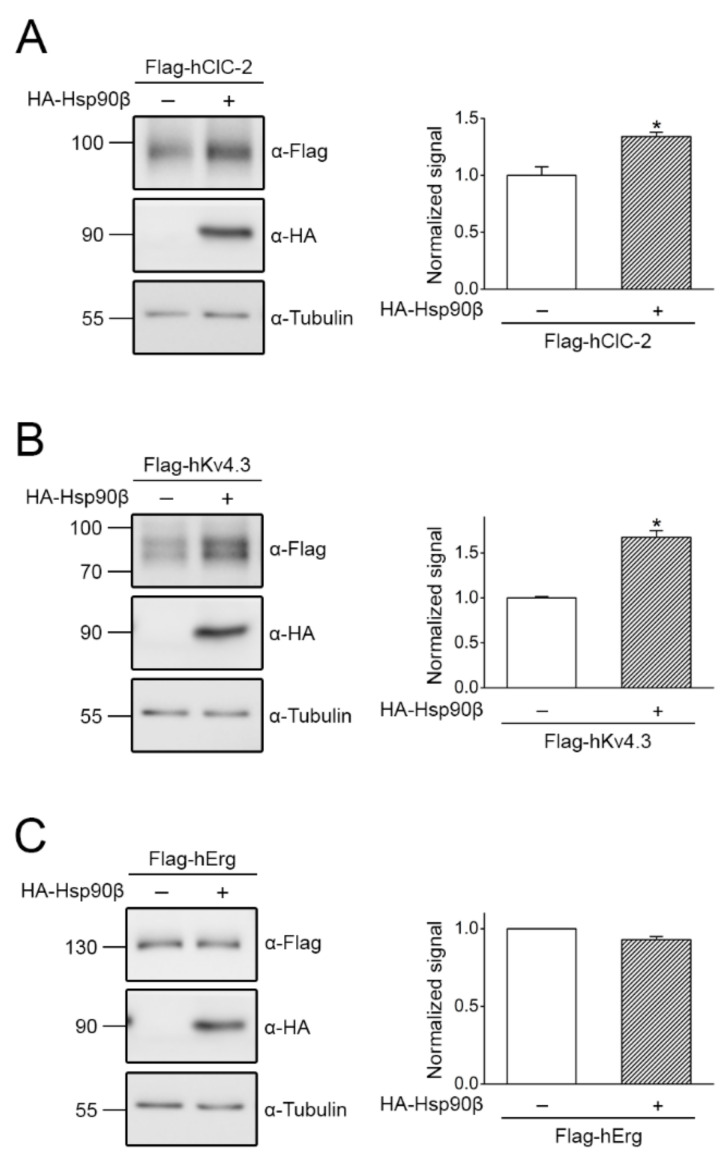
The effect of Hsp90β co-expression on human ClC-2, K_V_4.3, and Erg channel protein levels in CHO cells. Heterologous expression of Flag-hClC-2 (**A**), Flag-tagged human K_V_4.3 (Flag-hK_V_4.3) (**B**), or Flag-tagged human Erg (Flag-hErg) (**C**) channels in CHO cells. (Left panels) Representative immunoblots showing the effect of HA-Hsp90β co-expression on total protein levels. cDNA for Flag-hClC-2/hK_V_4.3/hErg was co-transfected with that for HA-Hsp90β in the molar ratio 1:3. Co-expression with the empty vector was employed as the control experiment. Tubulin was used as the loading control. (Right panels) Quantification of relative total protein levels in response to HA-Hsp90β co-expression. Protein density was standardized as the ratio of the ClC-2/K_V_4.3/Erg signal to the cognate tubulin signal. Values from the HA-Hsp90β co-expression groups (hatched bars) were then normalized to those for the corresponding vector control (clear bars). Asterisks denote significant difference from the vector control (*, *t* test: *p* < 0.05; *n* = 3).

We also verified the effect of Hsp90β co-expression on human ClC-2 heterologously expressed in CHO cells. Figure 5A illustrates that, similar to the result observed in HK293T cells, Hsp90β notably enhanced human ClC-2 protein level in CHO cells by about 34%. Hsp90β co-expression also prominently increased the total protein level of the human voltage-gated potassium channel subtype 4.3 (K_V_4.3) by about 68% (Figure 5B). In contrast, Hsp90β failed to discernibly affect the protein expression of the human Ether-à-go-go-related gene (Erg) channel (Figure 5C).

### 2.3. Pharmacological Promotion of ClC-2 Proteostasis with Hsp90-Targeting Small Molecule

**Figure 6:**
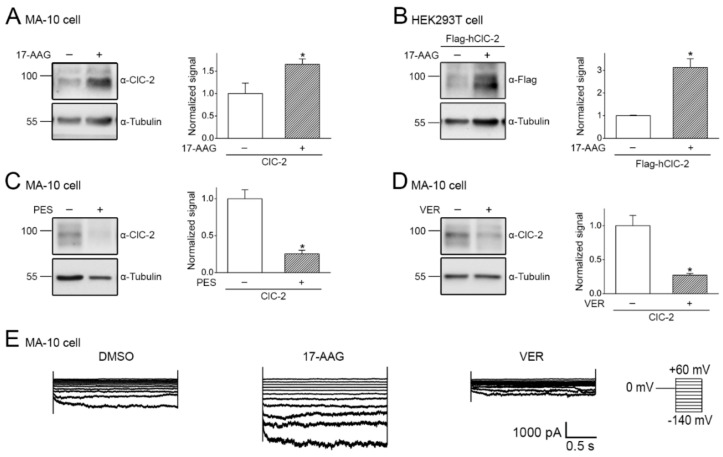
17-AAG enhances total ClC-2 protein expression. (**A**,**B**) The effect of 24-h treatment of 1 μM 17-AAG (in 0.1% DMSO) on endogenous ClC-2 expression in mouse MA-10 cells (**A**), as well as heterologous expression of human ClC-2 in HEK293T cells (**B**). (Left panels) Representative immunoblots. DMSO treatment was employed as the control experiment. Tubulin was used as the loading control. (Right panels) Quantification of relative ClC-2 protein levels in response to 17-AAG treatment. Protein density was standardized as the ratio of the ClC-2 signal to the cognate tubulin signal. Values from the 17-AAG treatment group (hatched bars) were then normalized to those for the corresponding DMSO control (clear bars). Asterisks denote significant difference from the DMSO control (*, *t* test: *p* < 0.05; *n* = 3–8). (**C**,**D**) The effect of 6-h treatment with 100 μM of 2-phenylethynesulfonamide (PES) (**C**) or VER-155008 (VER) (**D**) (in 0.1% DMSO) on endogenous ClC-2 protein expression in mouse MA-10 cells. (Left panels) Representative immunoblots. (Right panels) Quantification of relative ClC-2 protein levels. Values from the PES/VER treatment group (hatched bars) were then normalized to those for the corresponding DMSO control (clear bars). Asterisks denote significant difference from the DMSO control (*, *t* test: *p* < 0.05; *n* = 3). (**E**) Representative endogenous ClC-2 Cl^−^ current traces in mouse MA-10 cells in the presence of DMSO, 17-AAG, or VER treatments. Whole-cell patch clamp recording was implemented as described in the Methods section. The voltage clamp protocol (the rightmost panel) comprises a holding potential at 0 mV, followed by 2-sec voltage pulses ranging from −140 to +60 mV in 20-mV increments.

The aforementioned data indicate that ClC-2 is a client protein of Hsp90β and its associated chaperone/co-chaperone system. Hsp90β is known to stabilize numerous types of client proteins, including E3 ubiquitin ligases that promote degradation of misfolded proteins [41,42,43]. For example, Hsp90β directly interacts with and is essential for stabilizing CUL4 [34,44], which serves as an essential component of the CUL4-DDB1-CRBN E3 ubiquitin ligase complex mediating ubiquitination and proteasomal degradation of ClC-2 channels [20]. Depending on the proteostatic role of Hsp90β in ER quality control, pharmacological suppression of Hsp90β activity may either enhance or reduce protein expression of its client proteins [34,45,46,47]. One of the well-known Hsp90-targeting small molecules is 17-allylamino-17-demethoxygeldanamycin (17-AAG), which represses Hsp90 ATPase activity by blocking ATP binding [48,49]. Interestingly, 17-AAG has been shown to effectively suppress endogenous CUL4 expression in human cells [34,46]. We therefore decided to assess the effect of 17-AAG treatment on ClC-2 protein level. Figure 6A depicts that 24-h treatment with 1 μM 17-AAG significantly enhanced endogenous ClC-2 protein expression in mouse MA-10 Leydig cells by about 66%. In addition, 17-AAG treatment led to a more than three-fold increase in human ClC-2 protein level in human-derived HEK293T cells (Figure 6B). In contrast, 6-h treatment with 100 μM of the Hsc70/Hsp70 inhibitors 2-phenylethynesulfonamide (PES) and VER-155008 resulted in more than 70% reduction in endogenous ClC-2 protein levels in mouse MA-10 cells (Figure 6C,D). Furthermore, electrophysiological analyses revealed that 17-AAG and VER-155008 significantly promoted and suppressed, respectively, functional expression of endogenous ClC-2 channels in mouse MA-10 cells (Figure 6E). Together these observations imply that, despite of the dual role of Hsp90β in facilitating both ClC-2 and CUL4 protein folding, suppression of Hsp90β ATPase activity with 17-AAG results in a dominant disruption of CUL4 protein stability, thereby promoting ClC-2 protein expression.

**Figure 7:**
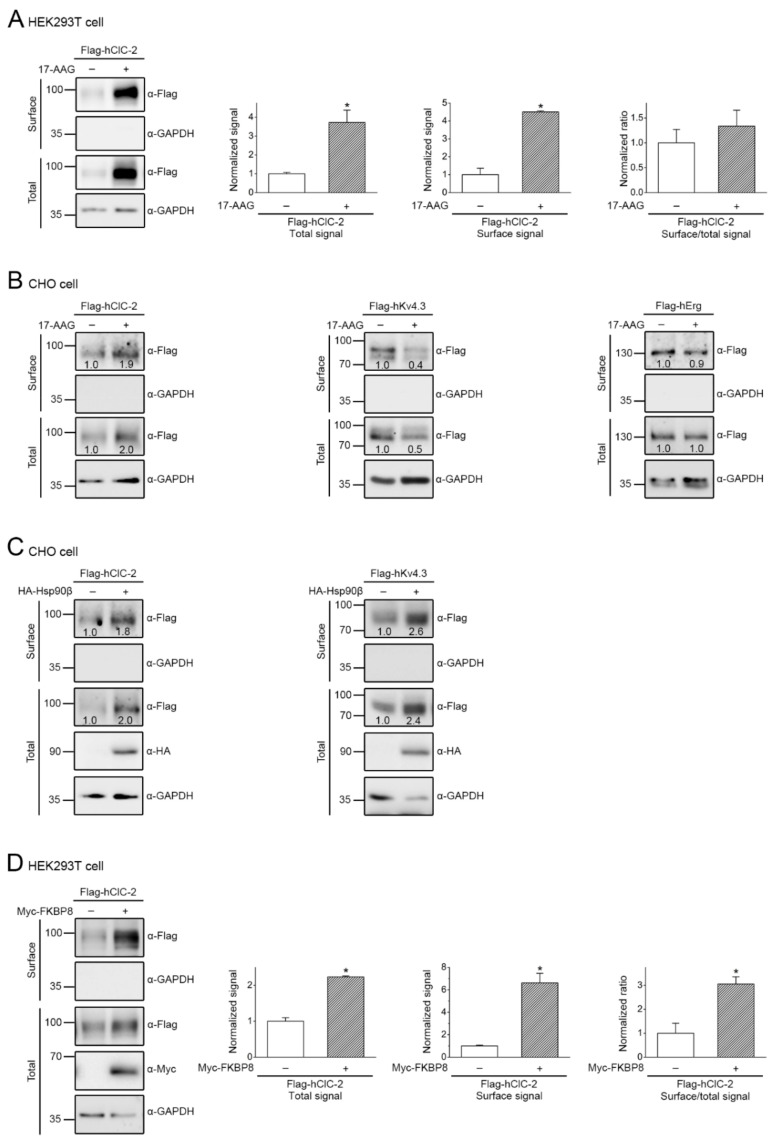
17-AAG, Hsp90β, and FKBP8 promote cell surface expression of human ClC-2 channels. (**A**) The effect of 24-h treatment of 1 μM 17-AAG (in 0.1% DMSO) on surface biotinylation experiments in HEK293T cells over-expressing human ClC-2. (Left panel) Representative immunoblots. Cell lysates from biotinylated intact cells were either directly employed for immunoblotting analyses (total) or subject to streptavidin pull-down prior to immunoblotting analyses (surface). GAPDH was used as the loading control. (Right panels) Quantification of surface protein level and surface expression efficiency (surface/total). The surface protein density was standardized as the ratio of surface signal to cognate total GAPDH signal, followed by normalization to that of the control (clear bars). The total protein density was standardized as the ratio of input signal to GAPDH signal. Surface expression efficiency was calculated as surface protein density divided by the corresponding total protein density, followed by normalization with respect to the surface-to-total ratio of the DMSO control (clear bars). Asterisks denote significant difference from the DMSO control (*, *t* test: *p* < 0.05; *n* = 4). (**B**,**C**) Representative immunoblots showing the effect of 17-AAG treatment (**B**) or Hsp90β co-expression (**C**) on surface biotinylation experiments in CHO cells over-expressing the indicated human ion channels. The numbers on the immunoblots denote the relative channel protein levels with respect to the control condition. (**D**) The effect of FKBP8 co-expression on surface biotinylation experiments in HEK293T cells over-expressing human ClC-2. (Left panel) Representative immunoblots. (Right panels) Quantification of surface protein level and surface expression efficiency. Asterisks denote significant difference from the vector control (*, *t* test: *p* < 0.05; *n* = 4).

So far, we have provided multiple lines of evidence showing that the Hsp90β-associated chaperone/co-chaperone system, as well as 17-AAG treatment, promotes total ClC-2 protein expression. Since ClC-2 serves as a plasma membrane Cl^−^ channel, it is important to determine whether cell surface ClC-2 protein level is effectively enhanced as well. By performing surface biotinylation analysis, we demonstrated that 17-AAG treatment resulted in nearly four-fold increase in both total and surface human ClC-2 protein levels in HEK293T cells (Figure 7A), indicating that the majority of 17-AAG-enhanced ClC-2 protein is properly folded at the ER and therefore effectively exported to the plasma membrane. Similarly in CHO cells, both 17-AAG treatment and Hsp90β co-expression increased total and surface human ClC-2 by about two-fold (Figure 7B,C). In comparison, 17-AAG substantially reduced total and surface human K_V_4.3 protein levels in CHO cells (Figure 7B), consistent with the idea that, unlike its dual role in the ER biogenesis of ClC-2, Hsp90β predominantly promotes K_V_4.3 protein expression (Figure 7C).

Interestingly, the comparable effect of 17-AAG and Hsp90β on total and cell surface ClC-2 protein expression (Figure 7A–C), as well as the absence of significant change in the surface-to-total protein density ratio, suggests that both 17-AAG and Hsp90β fail to detectably modify the membrane trafficking efficiency of ClC-2. Likewise, co-expression with Hsc70, HOP, or Aha1 promoted both total and surface ClC-2 protein level to a similar extent (data not shown), indicating a lack of measurable change in membrane trafficking efficiency. Nevertheless, FKBP8 co-expression resulted in about two-fold and six-fold increases in total and surface ClC-2, respectively (Figure 7D). This significant enhancement of the surface-to-total ClC-2 protein density ratio implies that, in addition to promoting protein folding at the ER, FKBP8 may also facilitate the membrane trafficking process of ClC-2 channels.

### 2.4. Correction of Disease-Related Defective ClC-2 Expression by 17-AAG

**Figure 8:**
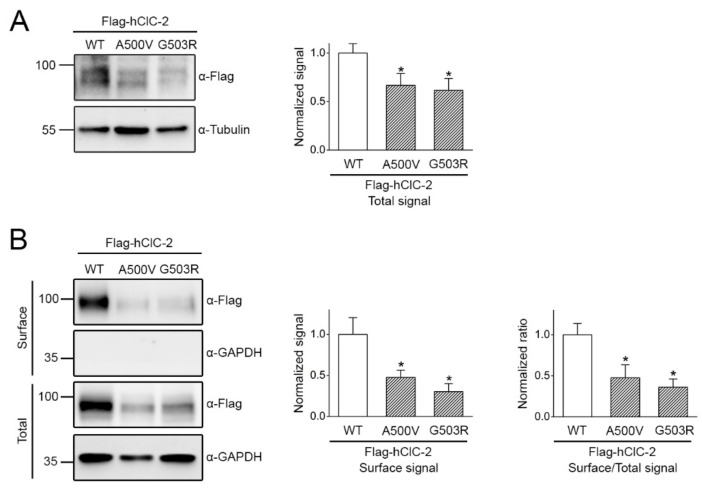
Leukodystrophy-causing mutant ClC-2 channels display reduced total and surface protein expressions, as well as impaired membrane trafficking efficiency. Human ClC-2 WT and leukodystrophy-associated A500V and G503R mutants were over-expressed in HEK293T cells. (**A**) (Left panel) Representative immunoblot comparing total protein level. (Right panel) Quantification of relative ClC-2 total protein level. Protein density was standardized as the ratio of the ClC-2 signal to the cognate tubulin signal. Values from the mutant groups (hatched bars) were then normalized to those for the corresponding WT control (clear bars). Asterisks denote significant difference from the WT (*, *t* test: *p* < 0.05; *n* = 5). (**B**) (Left panel) Representative immunoblot comparing surface protein level. (Right panels) Quantification of relative ClC-2 surface protein level and surface expression efficiency (surface/total). The surface protein density was standardized as the ratio of surface signal to cognate total GAPDH signal, followed by normalization to that of the WT control (clear bars). The surface expression efficiency was calculated as surface protein density divided by the corresponding total protein density, followed by normalization with respect to the surface-to-total ratio of the corresponding WT control (clear bars). Asterisks denote significant difference from the WT (*, *t* test: *p* < 0.05; *n* = 5).

**Figure 9:**
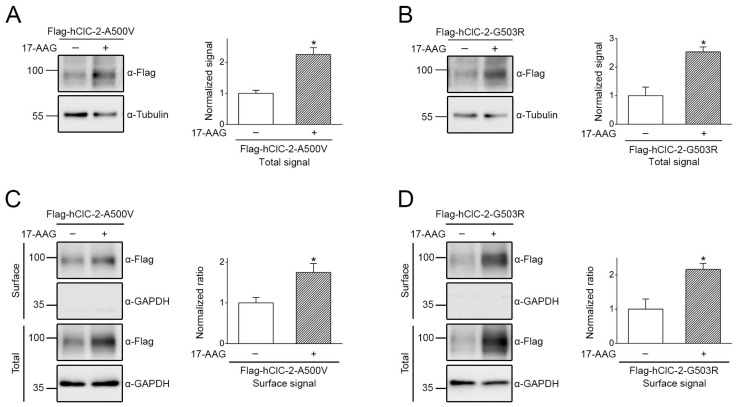
17-AAG ameliorates defective protein expression of human ClC-2 mutants. The effect of 17-AAG treatment on total (**A**,**B**) and surface (**C**,**D**) protein levels of the human ClC-2 A500V and G503R mutants. Heterologous expression of leukodystrophy-associated mutant human ClC-2 proteins in HEK293T cells was subject to 24-h treatment of 1 μM 17-AAG (in 0.1% DMSO). DMSO treatment was employed as the control experiment. (Left panels) Representative immunoblot. (Right panels) Quantification of relative ClC-2 protein level. Protein density was standardized as the ratio of the ClC-2 signal to the cognate tubulin signal. Values from the 17-AAG treatment group (hatched bars) were then normalized to those for the corresponding DMSO control (clear bars). Asterisks denote significant difference from the DMSO control (*, *t* test: *p* < 0.05; *n* = 4–8).

Two mutations located at the membrane-passing helix O of the human ClC-2 channel, A500V and G503R, have been linked to leukodystrophy [11,13,14]. Figure 8A illustrates that, compared to their WT counterpart, the total protein level of the two mutants was significantly decreased, consistent with the presence of anomalous proteostasis in disease-causing ClC-2 mutant channels. Surface biotinylation analyses further indicated that the ClC-2 A500V and G503R mutants were associated with substantially reduced cell surface expression, as well as notable defects in membrane trafficking efficiency (Figure 8B). Importantly, in response to 17-AAG treatment, the total protein level of the two leukodystrophy-causing ClC-2 mutants was enhanced by about 2-fold (Figure 9A,B). Moreover, 17-AAG substantially increased cell surface expression of the mutants by 75% or more (Figure 9C,D), indicating that a notable fraction of 17-AAG-rescued ClC-2 A500V and G503R proteins can be successfully transported from the ER to the plasma membrane.

## 3. Discussion

**Figure 10:**
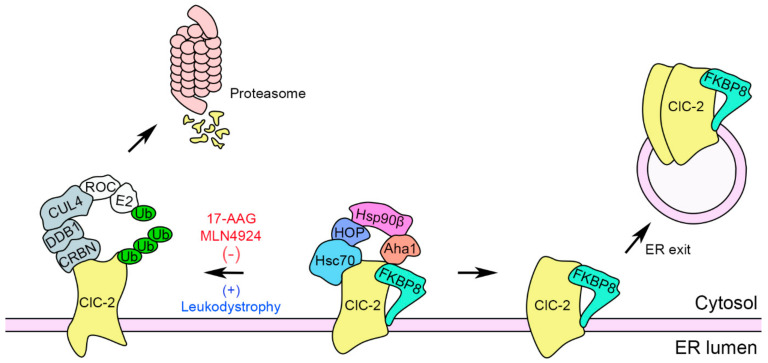
Schematic model of the ER quality control system for ClC-2 channels. In this schematic diagram of ClC-2 protein biogenesis at the ER, protein folding is primarily promoted by the constitutively expressed chaperones Hsp90β and Hsc70, as well as the co-chaperones HOP, Aha1, and FKBP8. FKBP8 may additionally contribute to a late stage of the ClC-2 protein folding process essential for subunit assembly, ER exit, and thereafter membrane trafficking. On the other hand, ER-associated degradation of ClC-2 is principally mediated by the scaffold protein CUL4 that forms a protein complex with the adaptor protein DDB1 and the substrate receptor protein CRBN. CUL4 also interacts with the RING-finger protein ROC, which recruits the E2 ubiquitin conjugating enzyme (E2) that transfers ubiquitin (Ub) for covalent linkage to a substrate protein. The CUL4-DDB1-CRBN E3 ubiquitin ligase complex catalyzes the ubiquitination of misfolded ClC-2 proteins. Ubiquitinated ClC-2 is subsequently targeted for proteasomal degradation. Leukodystrophy-causing mutations may instigate substantial protein misfolding that leads to increased ClC-2 protein degradation. In contrast, the Hsp90 inhibitor 17-AAG, as well as the cullin E3 ligase inhibitor MLN4924, significantly attenuates protein ubiquitination of ClC-2 channels, resulting in enhanced total and surface ClC-2 protein levels.

In this study, we aim to ascertain the molecular nature of the ER quality control system for ClC-2 Cl^−^ channels. As summarized in the schematic model illustrated in Figure 10, we propose that ClC-2 protein biogenesis at the ER is promoted by the Hsp90β-Hsc70 chaperone system associated with the co-chaperones HOP, Aha1, and FKBP8. In contrast, leukodystrophy-causing mutations tend to disrupt ClC-2 protein folding at the ER, leading to enhanced ClC-2 polyubiquitination by the CUL4-DDB1-CRBN E3 ubiquitin ligase complex and the ensuing proteasomal degradation [20].

Both our shRNA knock-down and chaperone over-expression data (Figure 3, Figure 4 and Figure 5) support the idea that the primary role of Hsp90β in ER quality control of ClC-2 involves a protein stabilization effect. However, Hsp90β also contributes to protein stabilization of CUL4 [34,44], the essential scaffold protein of the CUL4-DDB1-CRBN E3 ligase promoting ER-associated degradation of ClC-2 [20]. In other words, Hsp90β may play a dual role in regulating ClC-2 proteostasis at the ER: (i) directly promoting ClC-2 protein expression, but (ii) indirectly enhancing ClC-2 degradation via stabilizing CUL4. Nonetheless, pharmacological interruption of Hsp90β function with 17-AAG results in significant up-regulation of total and surface ClC-2 protein levels (Figure 6 and Figure 7), suggesting that 17-AAG may exert a dominant inhibition of the molecular chaperone’s effect on CUL4 protein stability. 17-AAG has been previously demonstrated to raise the protein level of another voltage-gated Cl^−^ channel, ClC-1 [34]. Interestingly, loss-of-function mutations in the human *CLCN1* gene encoding ClC-1 are associated with the skeletal muscle disease myotonia congenita and manifest aberrant proteostasis and gating of the Cl^−^ channel [5,50]. Both ClC-1 and ClC-2 belong to the CLC channel/transporter superfamily that includes three additional types of Cl^−^ channels [5,51,52]. Moreover, similar to the proteostatic mechanism of ClC-2, the CUL4-DDB1-CRBN E3 ligase complex promotes ER-associated degradation of the ClC-1 channel [53], and the Hsp90β-Hsc70 chaperone/co-chaperone system works in concert to promote ClC-1 protein folding at the ER [34]. It remains to be determined whether suppressing the ATPase function of Hsp90β with 17-AAG may facilitate ER proteostasis for the other members of the CLC channel/transporter superfamily as well.

In normal and stressed conditions alike, the constitutively expressed Hsc70 chaperone is essential for ER proteostasis, maintaining both protein folding and degradation [54,55]. For example, Hsc70 has been implicated in hereditary ion channel diseases such as cystic fibrosis and long QT syndrome, wherein Hsc70 partially contributes to protein folding at the ER but plays a critical role in promoting ER-associated degradation of disease-causing mutant ion channels [55,56]. Of note, Hsc70 and Hsp90 are also known to critically promote protein degradation and folding/trafficking, respectively, of steroid receptors [57,58,59]. In comparison, the primary role of Hsc70 in ER quality control of WT and disease-related ClC-1/ClC-2 channels is facilitation of protein folding (Figure 3 and Figure 4) [34]. One potential explanation for this apparent contrast in the functional character of Hsc70 in protein biogenesis may concern the molecular nature of the E3 ubiquitin ligase coupled with the molecular chaperone system: the former example involves carboxyl terminus Hsc70-interacting protein (CHIP), and the latter Hsp90β-interacting CUL4-DDB1-CRBN complex. Further investigation is required to determine whether and how E3 ubiquitin ligase is coupled with the proteostatic role of Hsc70 in protein folding and degradation.

As highlighted in the biotinylation data in Figure 7D, as well as the schematic diagram in Figure 10, FKBP8 is unique among the three identified ClC-2-interacting co-chaperones in that it promotes protein folding at the ER, as well as assisting membrane trafficking of ClC-2 channels. This observation implies that FKBP8 may additionally contribute to a late-stage ER protein folding process essential for ER exit and subsequent membrane trafficking. Importantly, FKBP8 also effectively facilitates membrane trafficking of the ClC-1 channel [34]. Moreover, FKBP8 may directly stabilize protein conformation of ClC-1 channels localized at the plasma membrane [60]. In contrast, despite its well established role in ER biogenesis, FKBP8 does not appear to contribute to trafficking and membrane stabilization of WT and mutant Cl^−^ channels implicated in cystic fibrosis [61]. It remains an open question whether the membrane trafficking effect of FKBP8 is specific for ClC-1 and ClC-2 channels only, or may be applicable to the other members of the CLC channel/transporter superfamily.

We report here that the Hsp90 inhibitor 17-AAG substantially increases total and cell surface protein levels of disease-associated loss-of-function ClC-2 mutant channels (Figure 9). Notably, 17-AAG has been extensively tested in a wide variety of different clinical trials as a tumor suppression drug [48,49,62], as well as a chemical chaperone for correcting anomalous proteostasis associated with neurodegenerative diseases [22,63,64]. In addition, we previously demonstrated that the CUL4 inhibitor MLN4924, an emerging anti-cancer drug [65,66,67,68,69], also effectively promotes protein expression of leukodystrophy-related ClC-2 mutant channels [20]. Taken together, as emphasized in the schematic model illustrated in Figure 10, our findings highlight the therapeutic potential of 17-AAG and MLN4924 as clinically applicable small molecules for correcting impaired ER proteostasis of leukodystrophy-causing, loss-of-function ClC-2 mutant proteins.

## 4. Materials and Methods

### 4.1. cDNA Constructs

Human ClC-2 cDNA (NM_004366) was subcloned into the pcDNA3-Flag vector (Invitrogen, Carlsbad, CA, USA) to generate the N-terminal Flag-tagged human ClC-2 (Flag-hClC-2) construct. Leukodystrophy-causing ClC-2 mutations (A500V and G503R) were generated by site-directed mutagenesis, followed by verification with DNA sequencing. Other cDNA constructs employed in this study include pcDNA3.1-Myc mouse Aha1, pcDNA3.1-Myc mouse FKBP8, pFlag-CMV2 human Erg, pcDNA3-HA rat HOP, pcDNA5-V5 human Hsc70 (Addgene 19514, Watertown, MA, USA), pcDNA3-HA human Hsp90β (Addgene 22847, Watertown, MA, USA), and pcDNA3-Flag human K_V_4.3.

### 4.2. Preparation of Animal Samples for Co-Immunoprecipitation

C57BL/6 mice were handled in accordance with the National Institute of Health Guide for the Care and Use of Laboratory Animals (NIH Publications No. 80-23, revised 1996, Bethesda, MD, USA). All procedures involving animals were performed in conformity with the animal protocol approved by the Institutional Animal Care and Use Committee (IACUC), College of Medicine, National Taiwan University.

Testes dissected from mice (about 6-weeks-old; weighing about 19 g) were homogenized in ice-cold T-PER tissue extraction reagent (Thermo Scientific, Waltham, MA, USA; 1 testis per 400 µL) containing protease inhibitor cocktail. Lysates were cleared by micro-centrifugation at 13,360× *g* for 15 min. Solubilized lysates were pre-cleared with protein G sepharose beads (GE Healthcare Biosciences, Piscataway Township, NJ, USA) for 2 h at 4 °C, and then incubated for 16 h at 4 °C with protein G sepharose beads pre-coated with rabbit IgG or rabbit anti-ClC-2 antibody. Beads were gently spun down and washed twice in a wash buffer [(in mM) 100 NaCl, 4 KCl, 2.5 EDTA, 20 NaHCO_3_, 20 Tris-HCl, pH 7.5] supplemented with 0.1% Triton X-100, and then twice with the wash buffer. The immune complexes were eluted from the beads by heating at 70 °C for 5 min in the Laemmli sample buffer.

### 4.3. Cell Culture and DNA Transfection

Mouse MA-10 Leydig cells and Chinese hamster ovary (CHO) cells were maintained in Dulbecco’s modified Eagle’s medium (DMEM)/F12 supplemented with 10% fetal bovine serum and 20 mM HEPES. HEK293T cells were grown in DMEM supplemented with 2 mM glutamine, 10% fetal bovine serum (Thermo Scientific, Waltham, MA, USA), 100 units/mL penicillin, and 50 μg/mL streptomycin, and were maintained at 37 °C in a humidified incubator with 5% CO_2_. Transient transfection in HEK293T and CHO cells was performed by using the Lipofectamine 2000 reagent (Invitrogen, Carlsbad, CA, USA). Cells were plated onto 12-well plates 24 hrs before transfection. Various expression constructs were incubated with the transfection reagent for 20 min at room temperature, and DNA-lipofectamine diluted in Opti-MEM (Invitrogen, Carlsbad, CA, USA) was added to culture wells. After 6-h incubation at 37 °C, the medium was changed and the culture cells were maintained in the 37 °C incubator for 48 hrs. Where indicated, 17-AAG, 2-phenylethynesulfonamide (PES; also known as pifithrin-µ), and VER-155008 (Sigma, St. Louis, MO, USA), dissolved in 0.1% dimethyl sulfoxide (DMSO), was applied to the culture medium.

### 4.4. RNA Interference

Lentivirus-based shRNA constructs (subcloned into the pLKO vector) targeting specific Aha1 (5′-CCCTGAGAAACATATTGTGAT-3′), FKBP8 (5′-AGTGGACATGACGTTCGAGGA-3′), ClC-2 (5′-CCTAGCTCCGAGACATCTATC-3′), HOP (5′-CGACCTTCATCAAGGGTTATA-3′), Hsc70 (5′-CGTCTGATTGGACGCAGATTT-3′), or Hsp90β (5′-CTTGTGTTGAAGGCAGTAAAC-3′) sequences, as well as the control shRNA for LacZ (5′-TGTTCGCATTATCCGAACCAT-3′) and GFP (5′-GACCACCCTGACCTACGGCGT-3′), were purchased from National RNAi Core Facility, Taiwan. Recombinant lentivirus was generated by co-transfecting HEK293T cells with the packaging plasmid pCMV-ΔR8.91, the envelope plasmid pMD.G, and shRNA expressing constructs via the jetPRIME transfection reagent (Polyplus-transfection, Illkirch, France). Then, 48 h after transfection, the medium was collected on a daily basis (stored at −80 °C; followed by application of new medium to HEK293T cells) for three consecutive days. The collected media containing lentiviral particles were centrifuged at 3000× *g* for 5 min, and supernatants were harvested and filtered (0.22 μm). MA-10 cells were maintained in the freshly collected viral supernatants in the presence of 8 μg/mL polybrene (Sigma, St. Louis, MO, USA) for at least 48 h, followed by incubation with a selection medium containing puromycin (5 µg/mL) for at least 48 h.

### 4.5. Immunoblotting

MA-10, HEK293T, and CHO cells were washed twice with ice-cold Dulbecco’s phosphate buffered saline (D-PBS) [(in mM) 137 NaCl, 2.7 KCl, 4.3 Na_2_HPO_4_·2H_2_O, 1.4 KH_2_PO_4_, pH 7.3] supplemented with 2 mM EDTA, and resuspended in a lysis buffer [(in mM) 150 NaCl, 5 EDTA, 50 Tris-HCl pH 7.6, 1% Triton X-100] containing a complete protease inhibitor cocktail (Roche, Basel, Switzerland). After adding the Laemmli sample buffer to the lysates, samples were sonicated on ice (three times for five seconds each) and heated at 70 °C for 5 min. Samples were then separated by 7.5–10% SDS-PAGE, electrophoretically transferred to nitrocellulose membranes, and detected using rabbit anti-Aha1 (1:2500; Thermo Scientific, Waltham, MA, USA), rabbit-anti-ClC-2 (1:1000; Alomone, Jerusalem, Israel), rabbit anti-Flag (1:5000; Sigma, St. Louis, MO, USA), rabbit anti-FKBP8 (1:4000; EnoGene, New York, NY, USA), rabbit anti-glyceraldehyde-3-phosphate dehydrogenase (GAPDH) (1:5000; GeneTex, Irvine, CA, USA), rat anti-HA (1:5000; Roche, Basel, Switzerland), rabbit anti-HOP (1:10000; Abcam, Cambridge, UK), rabbit anti-Hsc70 (1:750; Abcam, Cambridge, UK), rabbit anti-Hsp90β (1:500; Abcam, Cambridge, UK), mouse anti-Myc (clone 9E10), or rabbit anti-α-tubulin (1:5000; GeneTex, Irvine, CA, USA) antibodies. Blots were then exposed to horseradish peroxidase-conjugated anti-mouse/rabbit IgG (1:5000; Jackson ImmunoResearch, West Grove, PA, USA) or goat anti-rat IgG (1:5000; Santa Cruz, Dallas, TX, USA), and revealed by an enhanced chemiluminescence detection system (Thermo Scientific, Waltham, MA, USA). Where necessary, horseradish peroxidase-conjugated EasyBlot anti-rabbit IgG (GeneTex, Irvine, CA, USA) was employed as the secondary antibody to obscure the signal associated with rabbit heavy or light immunoglobulin chains. Acquisition of chemiluminescent signals from immunoblots was achieved by using the UVP AutoChemi image system (Ultra-Violet Products, Upland, CA, USA). Results shown are representative of at least three independent experiments.

For quantitative analyses, data were collected from at least three independent experiments performed in duplicates or triplicates. Densitometric scans of immunoblots were quantified by using ImageJ (National Institute of Health, Bethesda, MD, USA). For a given immunoblot containing multiple lanes of protein signals associated with the same experimental condition addressing a specific issue, protein density was first standardized as the ratio of the densitometric signal of the protein of interest to that of the cognate loading control. Standardized protein density values of all the control groups were then used for calculating the mean control protein density. Standardized protein density values of individual treatment or control groups were subsequently normalized with respect to the mean control protein density. For a given experimental condition with multiple repeats, normalized protein density values from multiple immunoblots were pooled together for statistical analyses.

### 4.6. Immunofluorescence

MA-10 cells grown on coverslips were rinsed in ice-cold phosphate buffered saline (PBS) and fixed with 4% paraformaldehyde at room temperature for 20 min, or with cold methanol at –20 °C for 10 min. After being washed with cold PBS, fixed cells were permeabilized and blocked with a blocking buffer (5% normal goat serum in 20 mM phosphate buffer, pH 7.4, 0.1% (*v*/*v*) Triton X-100, and 0.45 M NaCl) for 60 min at 4 °C. Cells were then immunolabeled overnight at 4 °C with 1:200 dilution (in the blocking buffer) of rabbit anti-Aha1, mouse anti-ClC-2 (Santa Cruz, Dallas, TX, USA), rabbit anti-FKBP8, rabbit anti-HOP, rabbit anti-Hsc70, or rabbit anti-Hsp90β antibodies. Alexa Fluor 488-conjugated anti-rabbit IgG and Alexa Fluor 568-conjugated anti-mouse IgG (1:200; Molecular Probes, Eugene, OR, USA) were used as secondary antibodies. Nuclei were stained with DAPI. After final wash, coverslips were mounted in a mounting medium (4% n-propylgallate, 90% glycerol, 0.1 M carbonate buffer, pH 9.2). Fluorescence images were viewed and acquired with a confocal microscope (TCS SP8, Leica, Wetzlar, Germany).

### 4.7. Electrophysiology

Conventional whole-cell patch clamp techniques were employed to record endogenous ClC-2 Cl^−^ currents in MA-10 cells. Cells were kept in the bath solution comprising (in mM): 140 NaCl, 4 CsCl, 2 MgCl_2_, 2 CaCl_2_, 10 HEPES, pH 7.4. Glass pipette electrodes with a resistance of 2–3 MΩ were filled with the intracellular solution containing (in mM): 120 CsCl, 10 EGTA, 10 HEPES, pH 7.4. Data were acquired and digitized with Axopatch 200A and Digidata 1322A, respectively, via pCLAMP 10 (Molecular Devices, San Jose, CA, USA). Cell capacitances were measured using a built-in function of pCLAMP 10 and were compensated electronically with Axopatch 200A. The holding potential was set at 0 mV. Data were sampled at 10 kHz and filtered at 1 kHz. All recordings were performed at room temperature (20–22 °C).

### 4.8. Cell Surface Biotinylation

HEK293T and CHO cells were rinsed with ice-cold D-PBS supplemented with 0.5 mM CaCl_2_, 2 mM MgCl_2_, incubated in 1 mg/mL sulfo-NHS-LC-biotin (Thermo Scientific, Waltham, MA, USA) in D-PBS at 4 °C for 1 h with gentle rocking on an orbital shaker, and subject to a quenching procedure by removing the biotin reagents and rinsing 3 times with 100 mM glycine in PBS, followed by once in Tris buffered saline [(in mM) 20 Tris-HCl, 150 NaCl, pH 7.4]. Cells were solubilized in ice-cold lysis buffer [(in mM) 150 NaCl, 50 Tris-HCl, 1% Triton X-100, 5 EDTA, 1 PMSF, pH 7.6] supplemented with protease inhibitor cocktail. Insolubilized materials were removed by centrifugation at 4 °C. Solubilized cell lysates were incubated overnight at 4 °C with streptavidin-agarose beads (Thermo Scientific, Waltham, MA, USA). Beads were washed once in the lysis buffer, followed by twice in a high-salt buffer [(in mM) 500 NaCl, 5 EDTA, 50 Tris-HCl, pH 7.6, 0.1% Triton X-100] and once in a low-salt buffer [(in mM) 2 EDTA, 10 Tris-HCl, pH 7.6, 0.1% Triton X-100]. Biotin-streptavidin complexes were eluted from the beads by heating at 70 °C for 5 min in the Laemmli sample buffer.

Cell lysates from biotinylated intact cells were subject to either direct immunoblotting analyses (regarded as total protein level; *total*) or streptavidin pull-down prior to immunoblotting analyses (regarded as surface protein level; *surface*). During sample loading for SDS-PAGE, the amount of lysates loaded in the *total* lane represents about 8% of that loaded in the *surface* lane. For quantitative analyses of *total* and *surface* signals, protein density was standardized as the ratio to the cognate loading control in the *total* lane. Surface expression efficiency (*surface/total*) was calculated as surface protein density divided by the corresponding standardized total protein density, followed by normalization with respect to the surface-to-total ratio of the control.

### 4.9. Statistical Analyses

All values were presented as mean ± SEM. Based on the assumption of normality and homogeneity of variance, the significance of the difference between two means was tested using the Student’s *t* test, whereas means from multiple groups were compared using the one-way ANOVA analysis, followed by post hoc analysis with the Bonferroni *t* test. All statistical analyses were performed with Origin 7.0 (Microcal Software, Northampton, MA, USA).

## Data Availability

The data that support the findings of this study are available from the corresponding authors, upon reasonable request.

## Abbreviations

17-AAG	17-allylamino-17-demethoxygeldanamycin
Aha1	activator of Hsp90 ATPase homolog 1
CHIP	carboxyl terminus Hsc70-interacting protein
Cl^−^	chloride
CHO	Chinese hamster ovary
CRBN	cereblon
CUL	cullin
DDB	damage-specific DNA binding protein
D-PBS	Dulbecco’s phosphate buffered saline
DMEM	Dulbecco’s modified Eagle’s medium
DMSO	dimethyl sulfoxide
DTT	dithiothreitol
ER	endoplasmic reticulum
Erg	Ether-à-go-go-related gene
FKBP8	FK506-binding protein 8
GAPDH	glyceraldehyde-3-phosphate dehydrogenase
HEK	human embryonic kidney
HOP	Hsp70/Hsp90 organizing protein
Hsc70	heat shock cognate protein 70
Hsp90β	heat shock protein 90β
IP	immunoprecipitation
K_V_4.3	voltage-gated potassium channel subtype 4.3
PBS	Phosphate buffered saline
PES	2-phenylethynesulfonamide
PMSF	phenylmethylsulfonyl fluoride
WT	wild-type
